# Supplementary material for: A Positive Regulatory Feedback Loop between EKLF/KLF1 and TAL1/SCL Sustaining the Erythropoiesis
Source: Int J Mol Sci. 2021 Jul 27;22(15):8024. doi: 10.3390/ijms22158024 (PMC8347936; doi:10.3390/ijms22158024)
Supplement: Supplementary file 1 [file ijms-22-08024-s001.zip › ijms-1297849-supplementary/ijms-1297849-supplementary.pdf]

LIST of SUPPLEMENTARY TABLES

Supplementary Table S1A. List of EKLF direct downstream targets generated from matching between the Affymetrix expression profiling comparing wild-type and Eklf$^{-/-}$ (KO) mice and the NimbleGen ChIP-chip promoter array.

Supplementary Table S1B. List of EKLF up-regulated genes after filtering with effect sizes.

Supplementary Table S1C. List of EKLF down-regulated genes after filtering with effect sizes.

Supplementary Table S2. List of EKLF-bound regions on mouse genome.

Supplementary Table S3A. List of top enriched network functions from up-regulated genes derived from Ingenuity IPA software.

Supplementary Table S3B. List of top enriched network functions from down-regulated genes derived from Ingenuity IPA software.

Supplementary Table S4A. List of molecular and cellular functions from up-regulated genes derived from Ingenuity IPA software.

Supplementary Table S4B. List of molecular and cellular functions from down-regulated genes derived from Ingenuity IPA software.

Supplementary Table S5A. List of significant canonical pathways from up-regulated genes derived from Ingenuity IPA software.

Supplementary Table S5B. List of significant canonical pathways from down-regulated genes derived from Ingenuity IPA software.

Supplementary Table S6. The occurrences of binding motifs of 226 transcription factors in EKLF-bound regions.

Supplementary Table S7. List of primers used in ChIP-chip data validation by ChIP- qPCR.

Supplementary Table S8. List of RT-PCR primers of used for validation of microarray hybridization data.

Supplementary Table S9. List of primers used for identification of Tal1 exon-1.
