# Supplementary material for: A Positive Regulatory Feedback Loop between EKLF/KLF1 and TAL1/SCL Sustaining the Erythropoiesis
Source: Int J Mol Sci. 2021 Jul 27;22(15):8024. doi: 10.3390/ijms22158024 (PMC8347936; doi:10.3390/ijms22158024)
Supplement: Supplementary file 1 [file ijms-22-08024-s001.zip › ijms-1297849-supplementary/sup-Fig_Edit.pptx]

## Slide 1
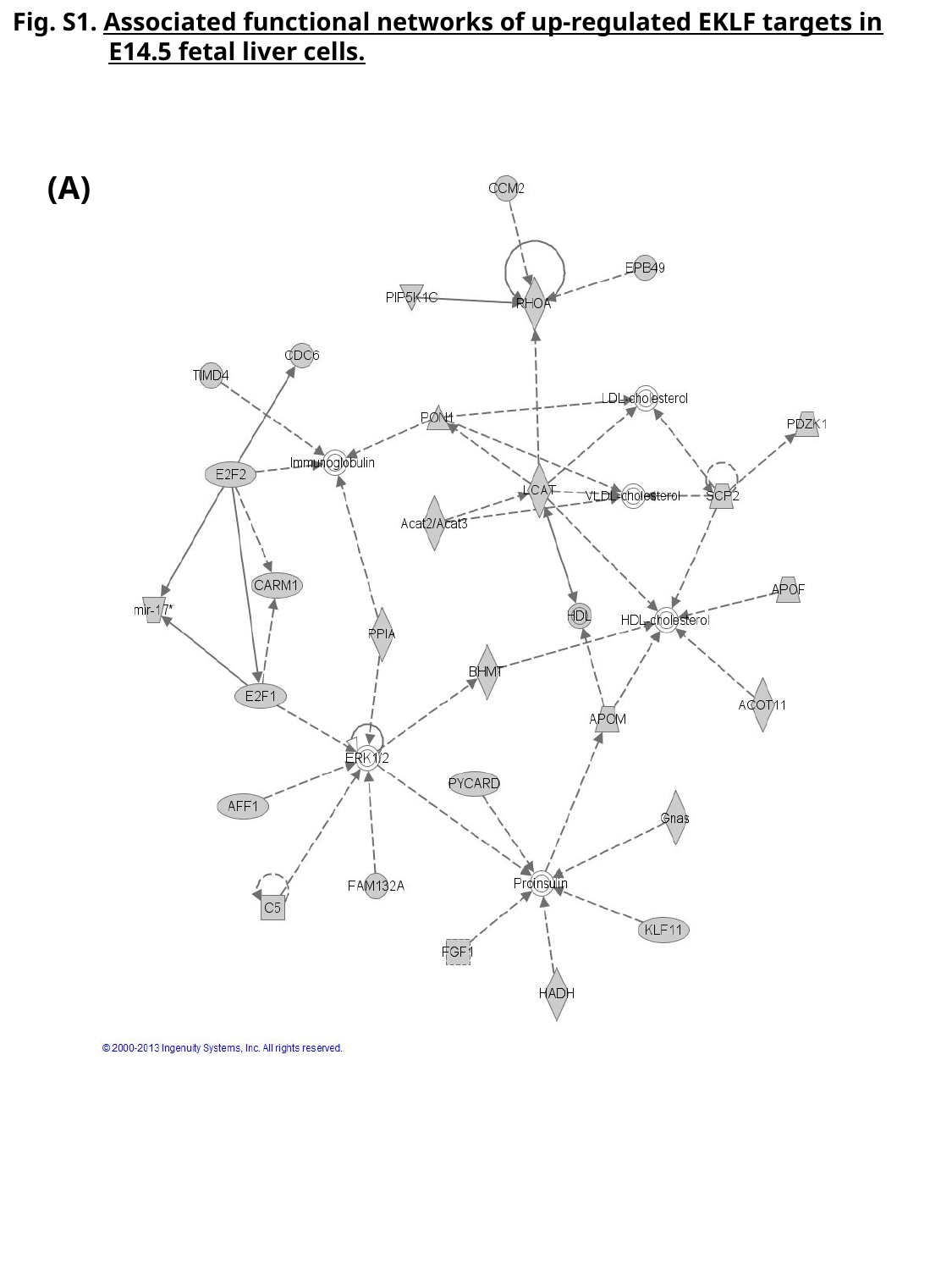

Fig. S1. Associated functional networks of up-regulated EKLF targets in E14.5 fetal liver cells.
(A)

## Slide 2
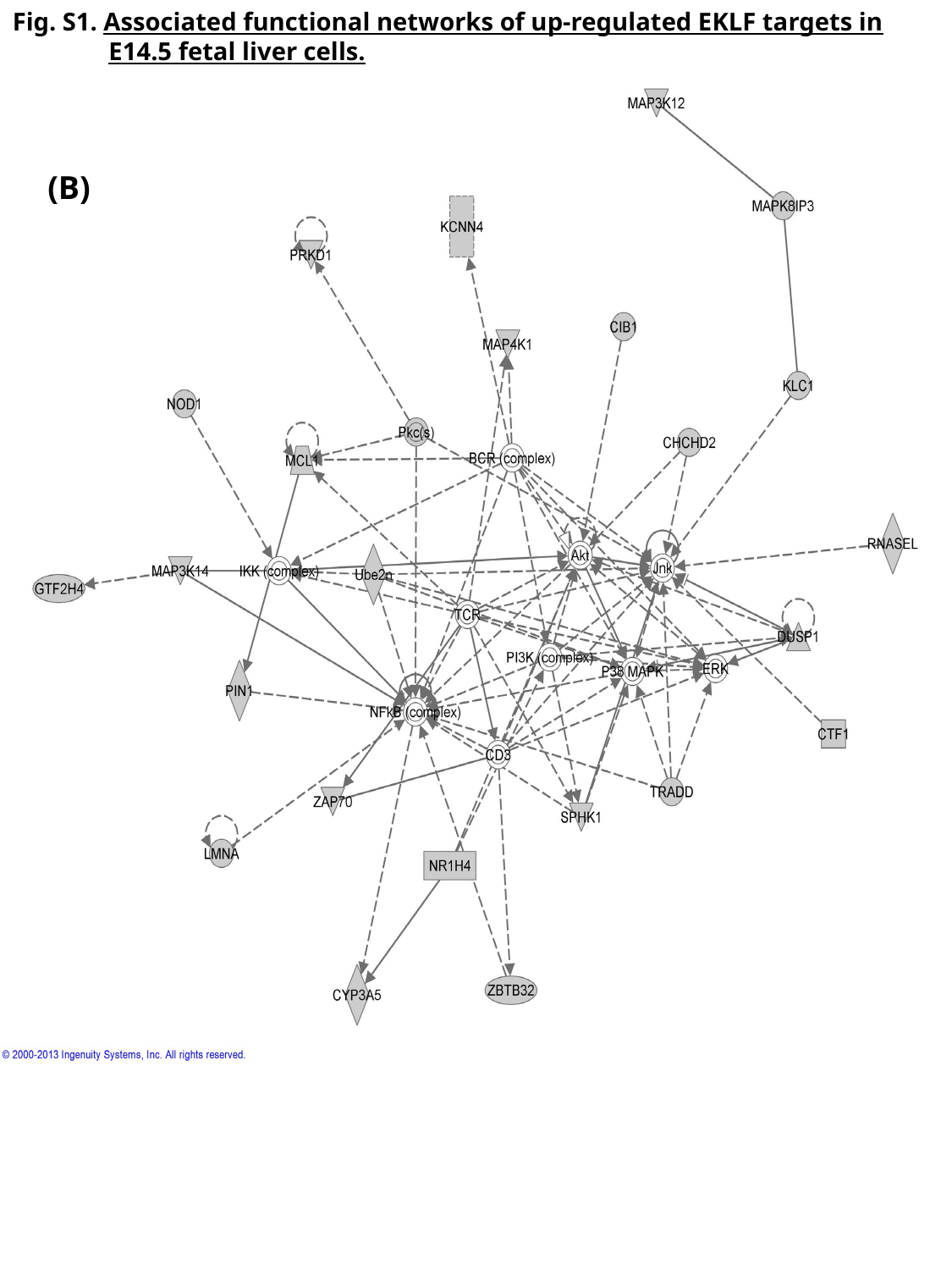

Fig. S1. Associated functional networks of up-regulated EKLF targets in E14.5 fetal liver cells.
(B)

## Slide 3
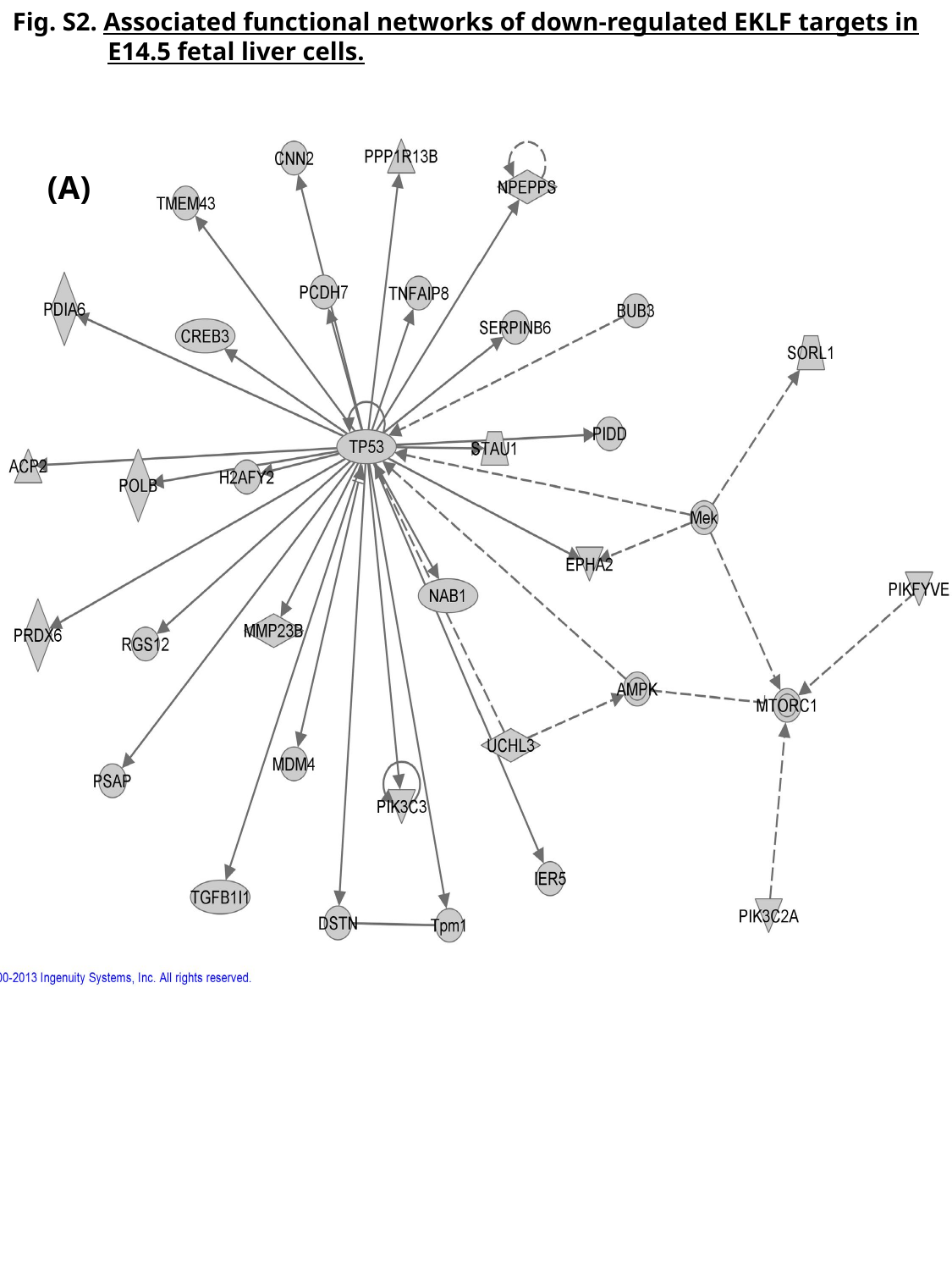

Fig. S2. Associated functional networks of down-regulated EKLF targets in E14.5 fetal liver cells.
(A)

## Slide 4
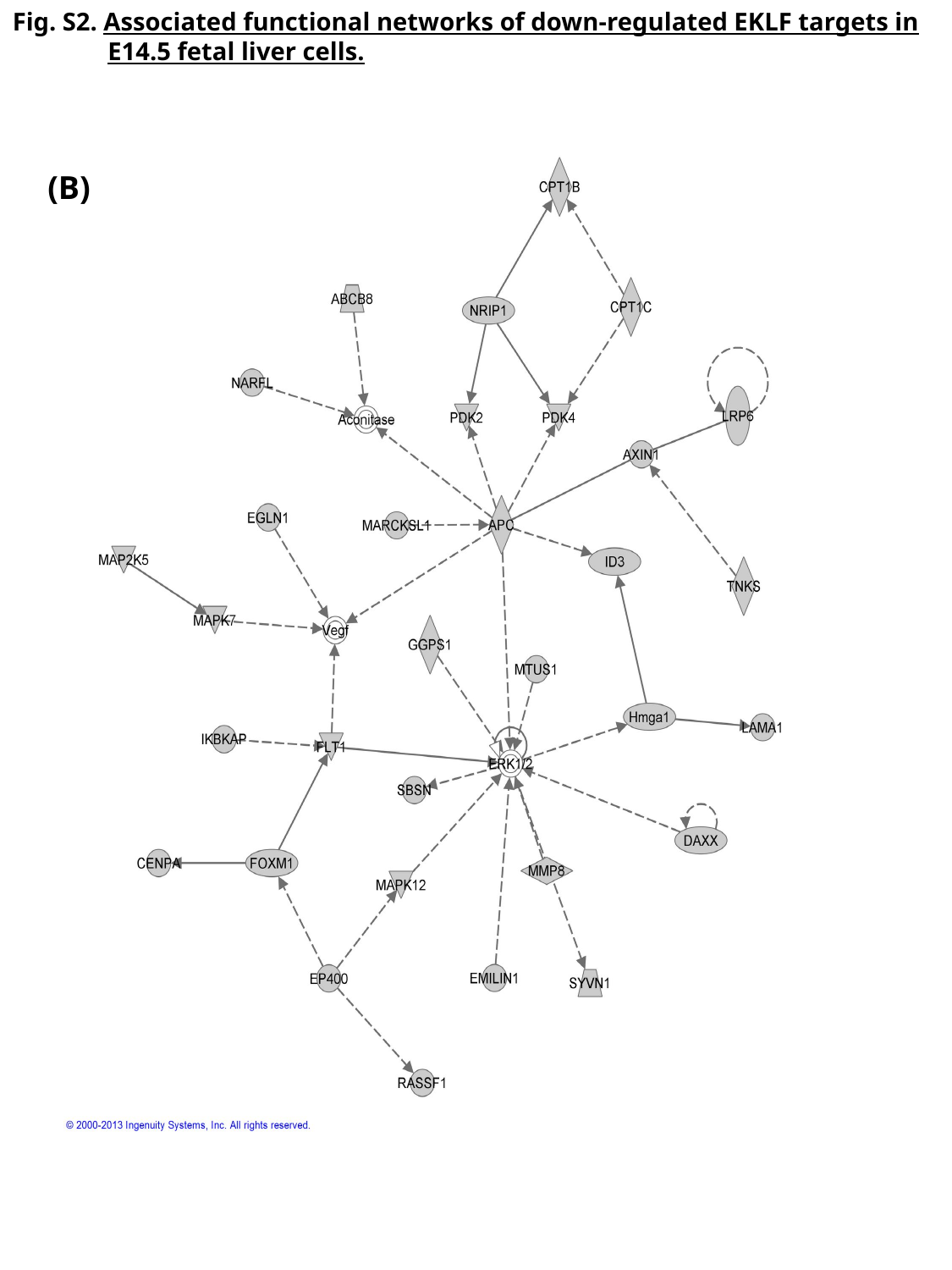

Fig. S2. Associated functional networks of down-regulated EKLF targets in E14.5 fetal liver cells.
(B)

## Slide 5
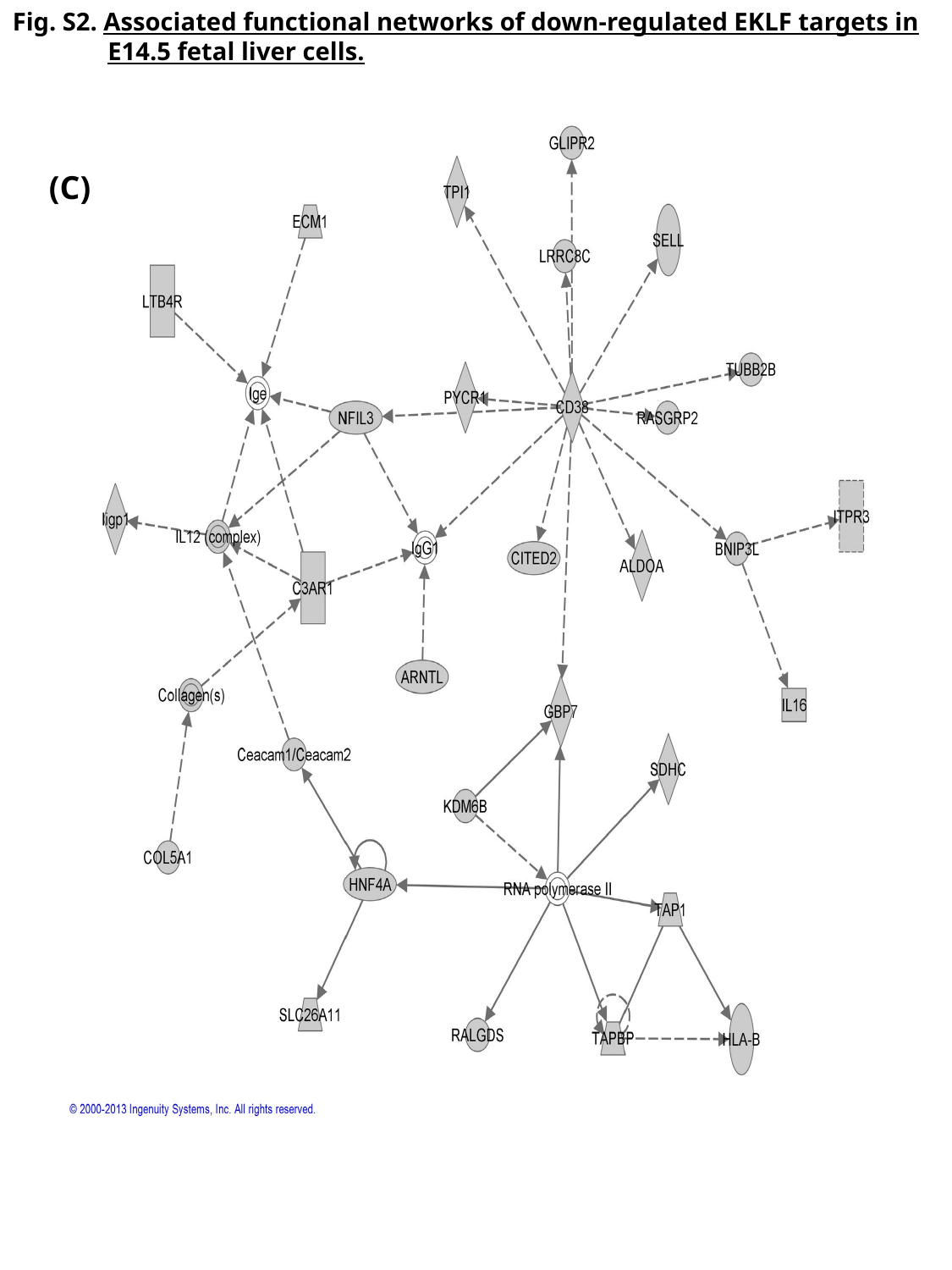

Fig. S2. Associated functional networks of down-regulated EKLF targets in E14.5 fetal liver cells.
(C)

## Slide 6
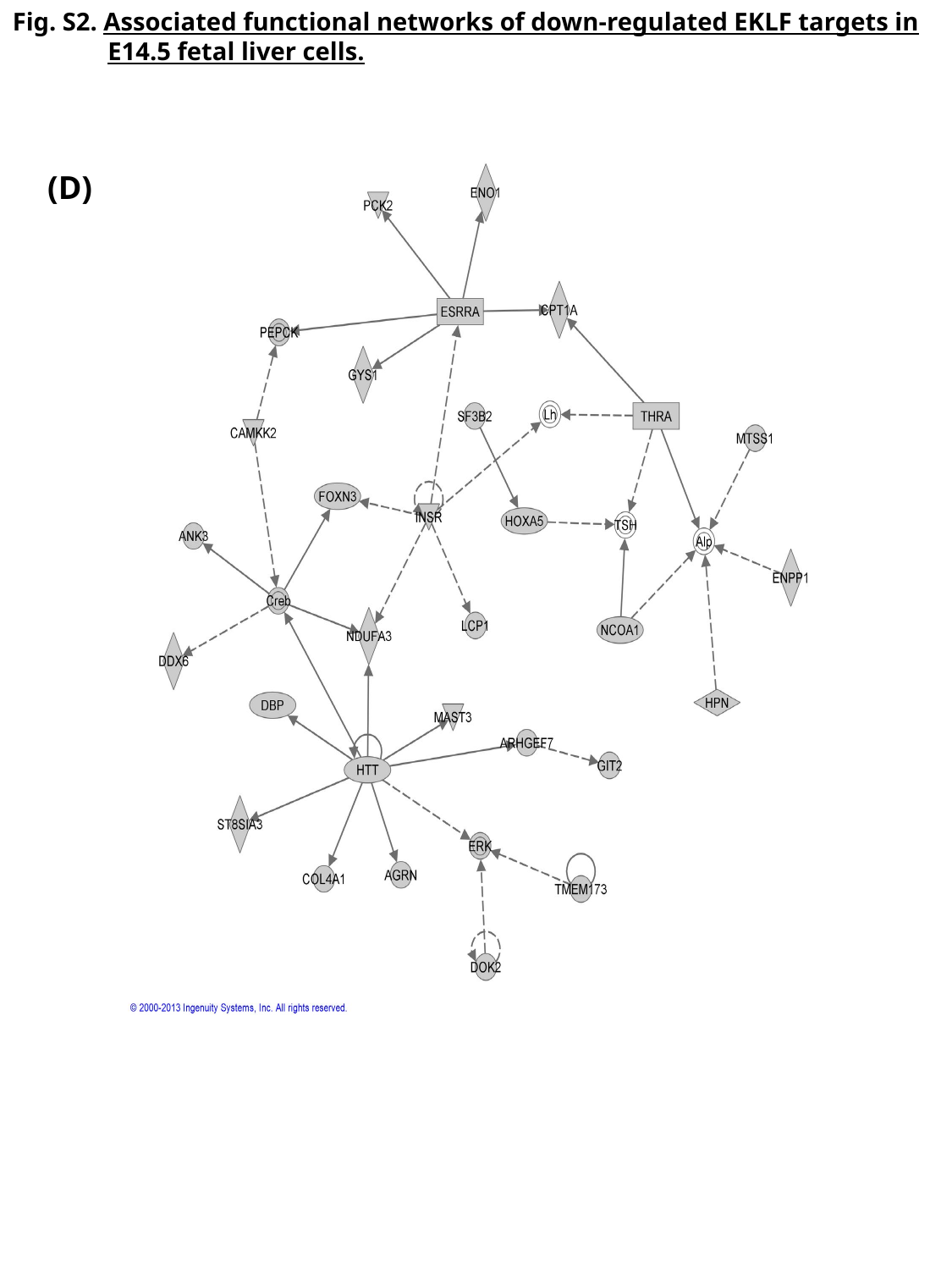

Fig. S2. Associated functional networks of down-regulated EKLF targets in E14.5 fetal liver cells.
(D)

## Slide 7
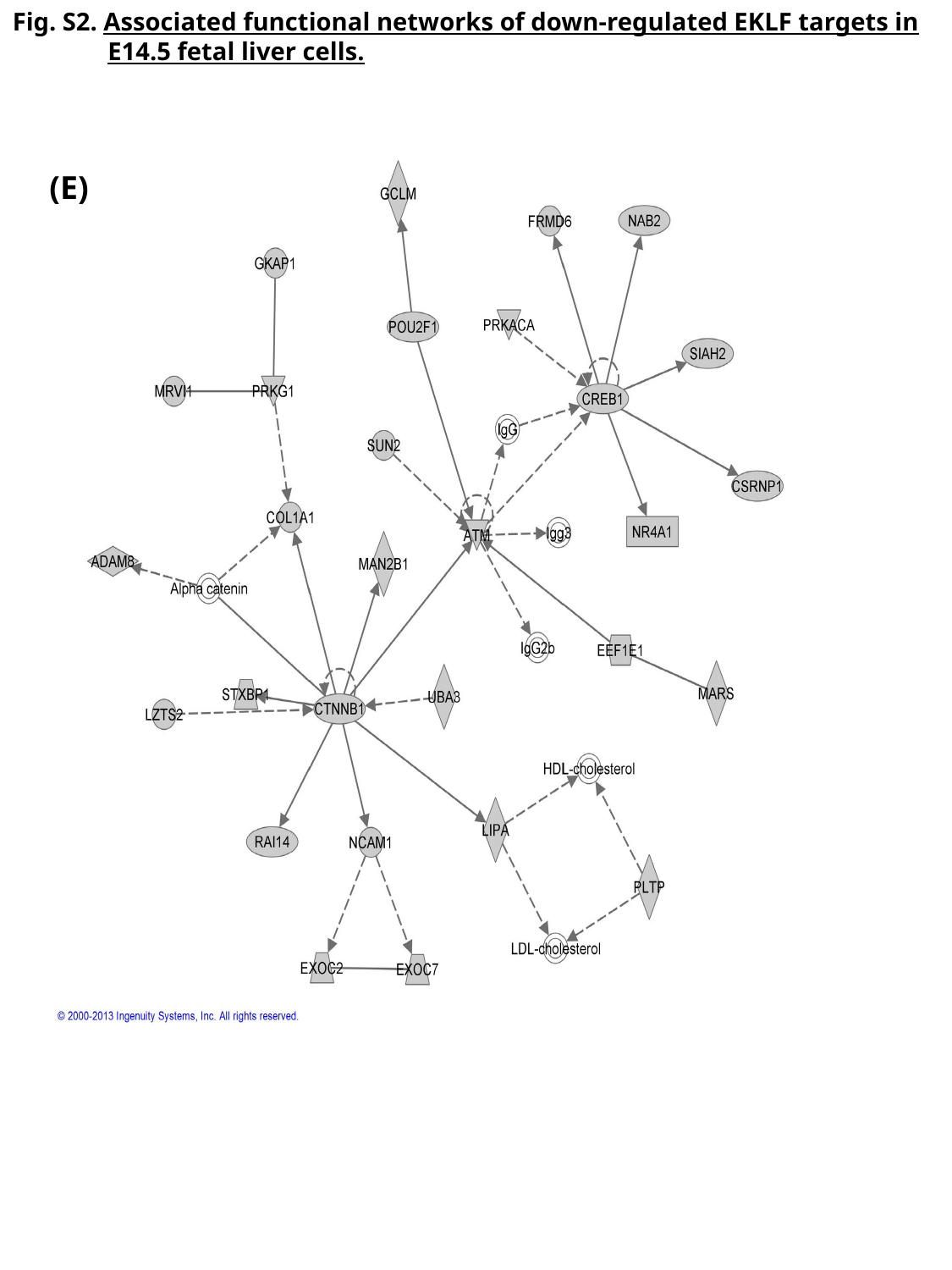

Fig. S2. Associated functional networks of down-regulated EKLF targets in E14.5 fetal liver cells.
(E)

## Slide 8
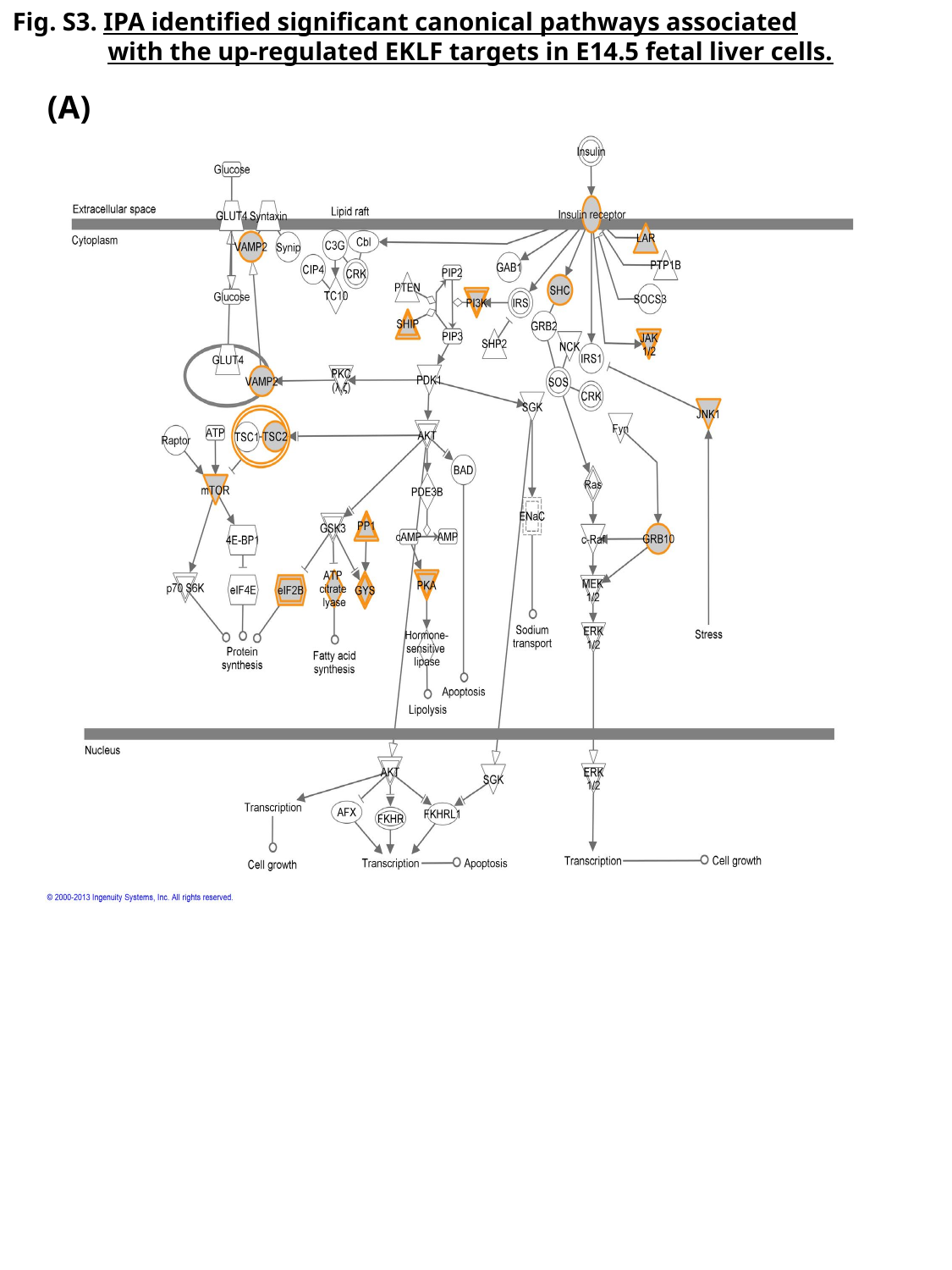

Fig. S3. IPA identified significant canonical pathways associated with the up-regulated EKLF targets in E14.5 fetal liver cells.
(A)

## Slide 9
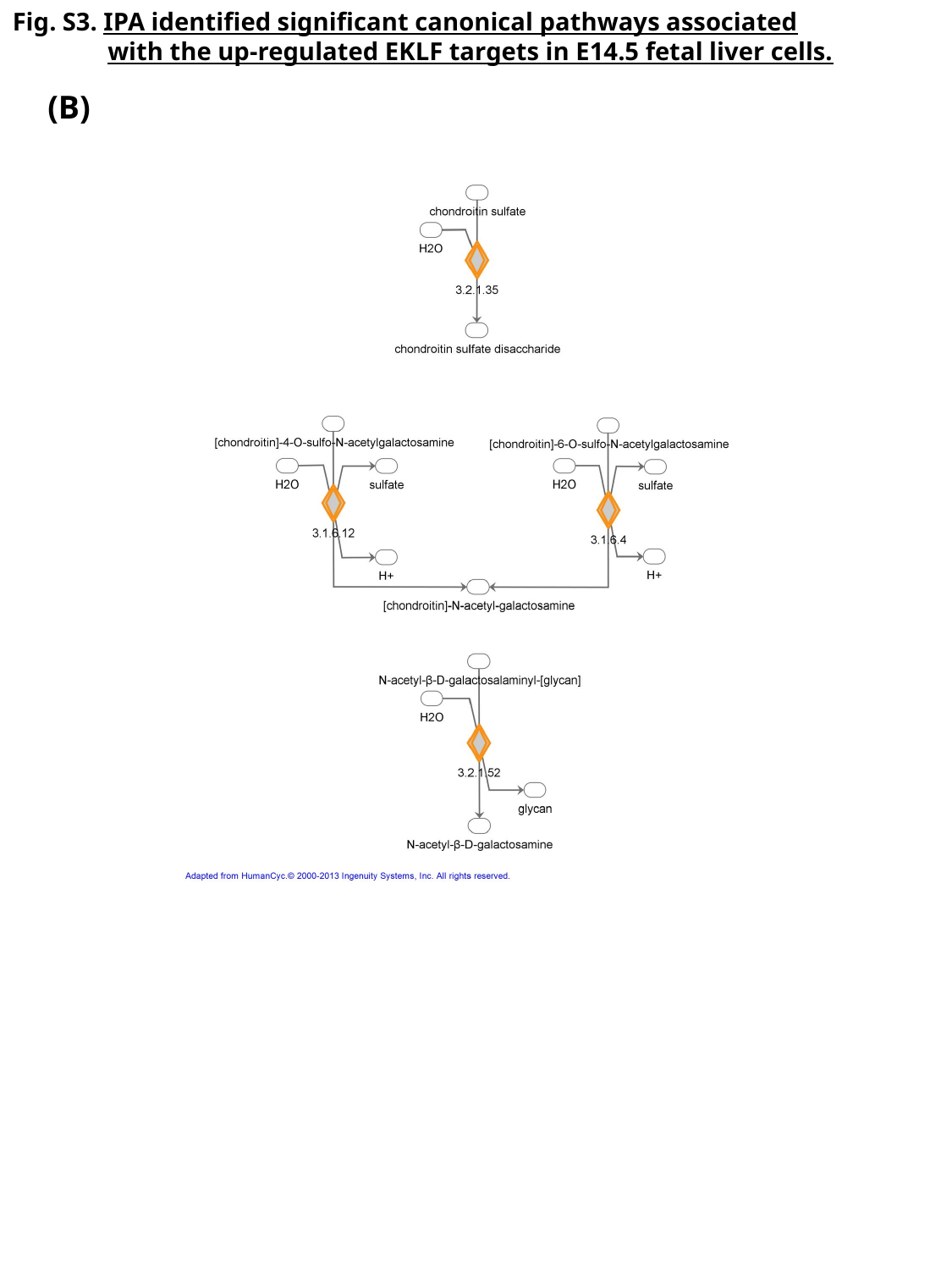

Fig. S3. IPA identified significant canonical pathways associated with the up-regulated EKLF targets in E14.5 fetal liver cells.
(B)

## Slide 10
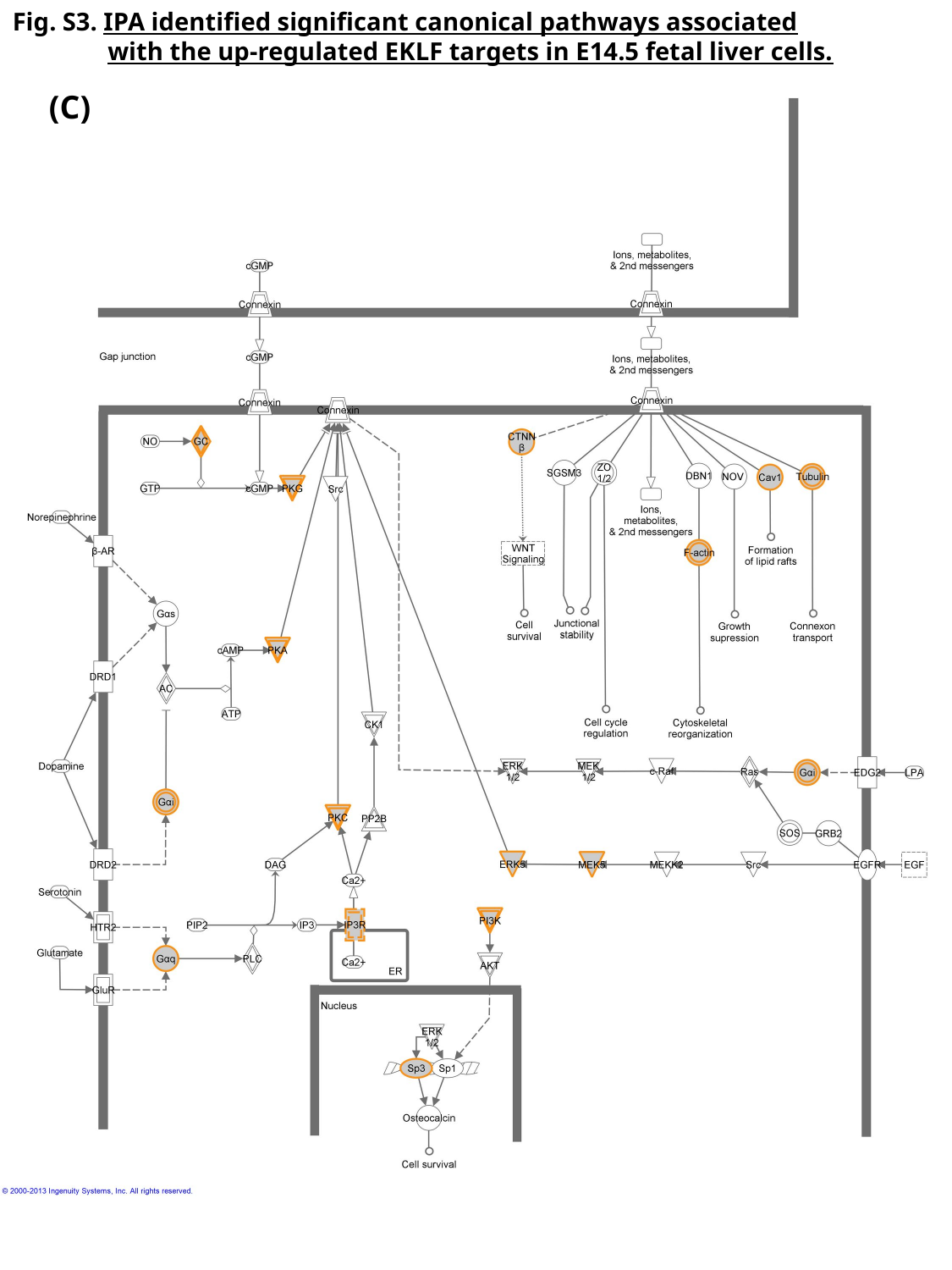

Fig. S3. IPA identified significant canonical pathways associated with the up-regulated EKLF targets in E14.5 fetal liver cells.
(C)

## Slide 11
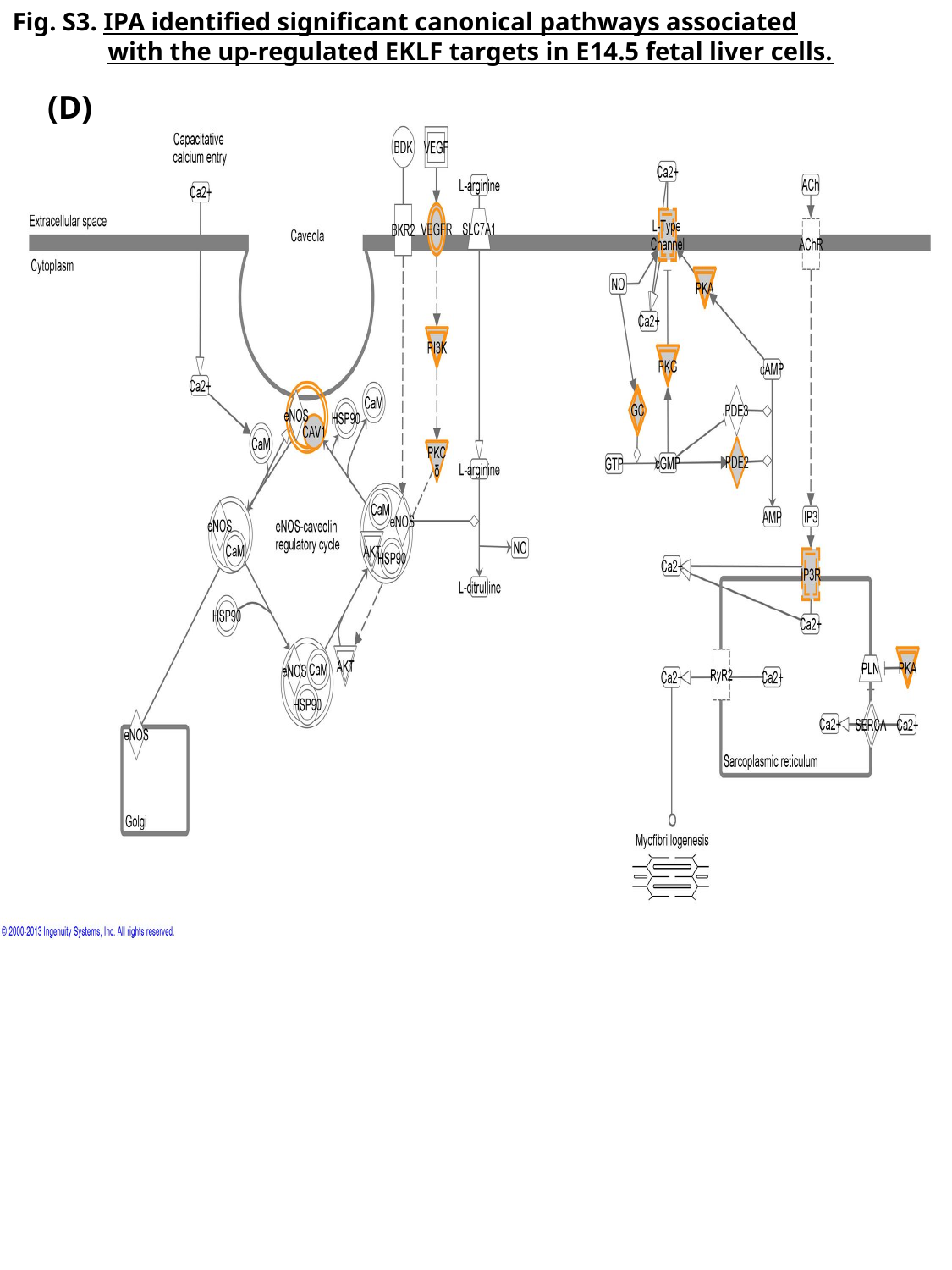

Fig. S3. IPA identified significant canonical pathways associated with the up-regulated EKLF targets in E14.5 fetal liver cells.
(D)

## Slide 12
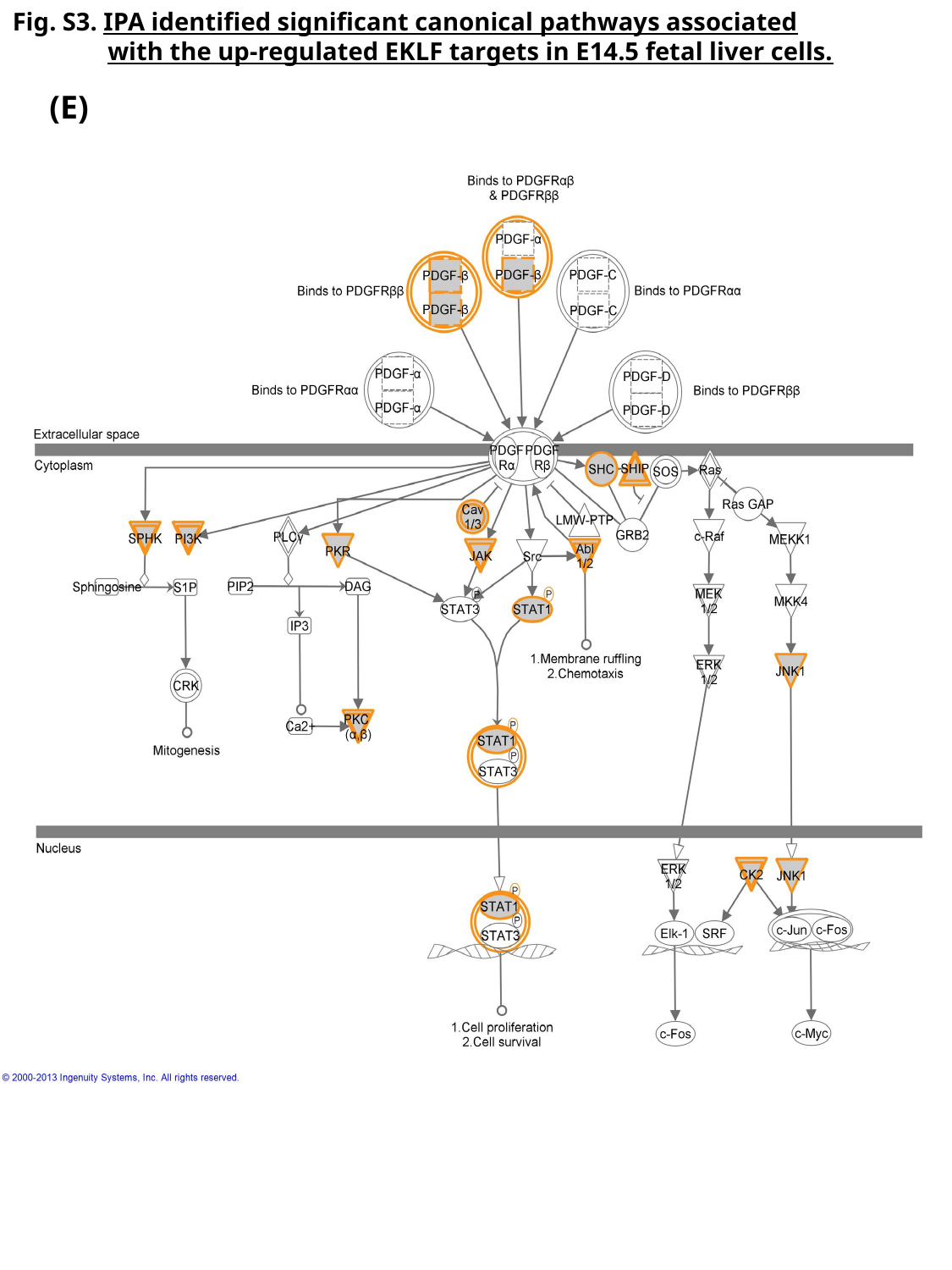

Fig. S3. IPA identified significant canonical pathways associated with the up-regulated EKLF targets in E14.5 fetal liver cells.
(E)

## Slide 13
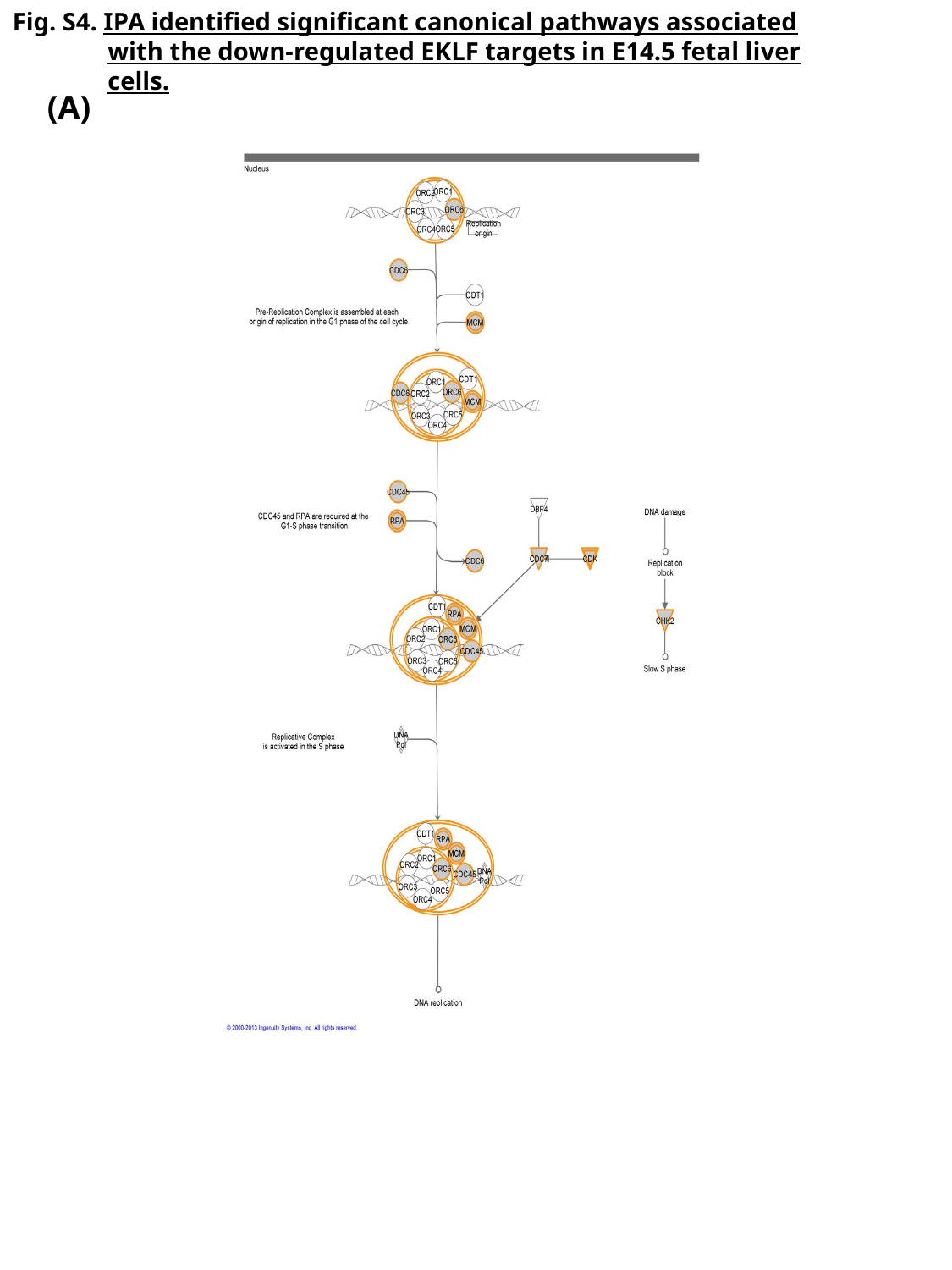

Fig. S4. IPA identified significant canonical pathways associated with the down-regulated EKLF targets in E14.5 fetal liver cells.
(A)

## Slide 14
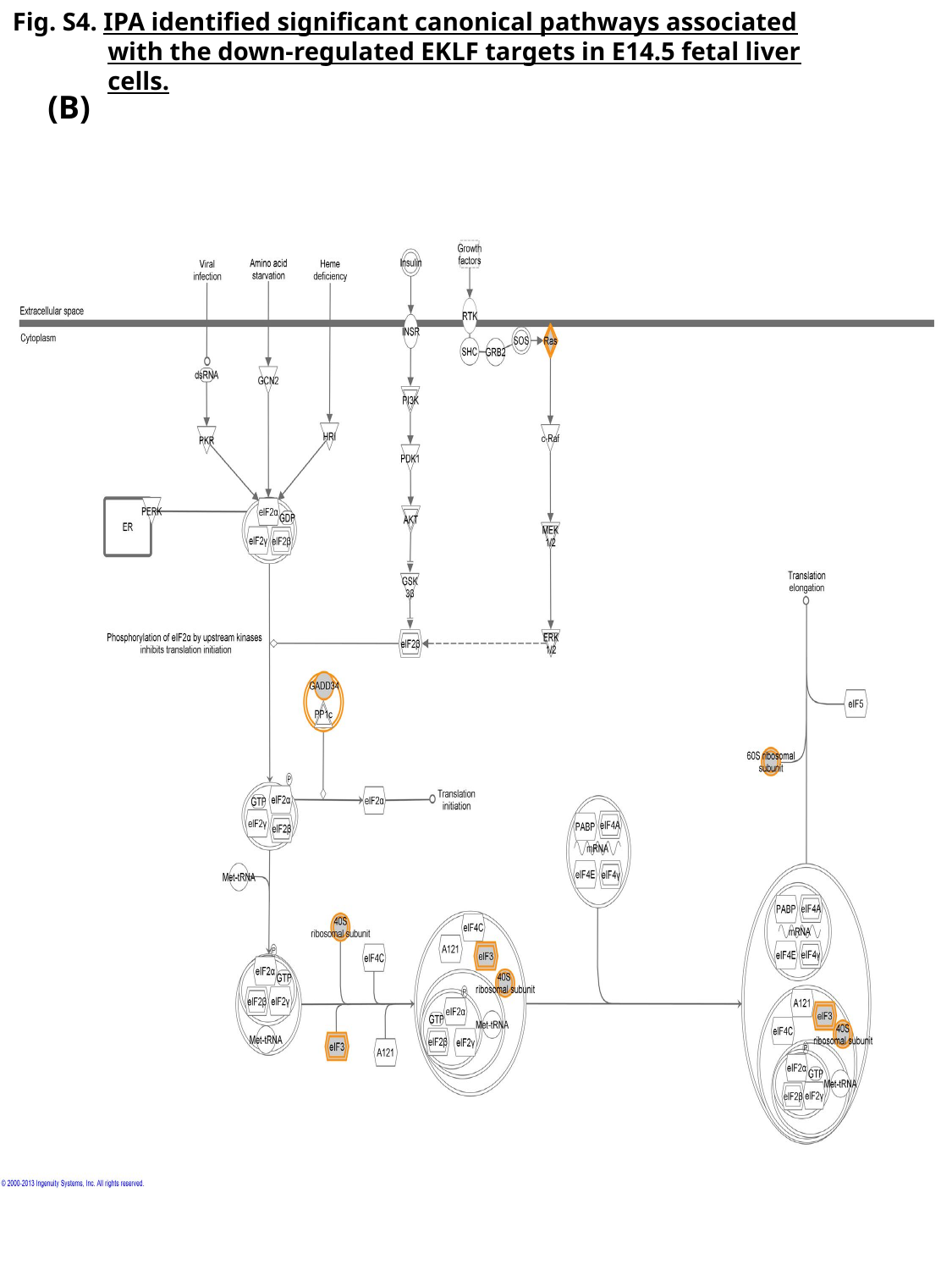

Fig. S4. IPA identified significant canonical pathways associated with the down-regulated EKLF targets in E14.5 fetal liver cells.
(B)

## Slide 15
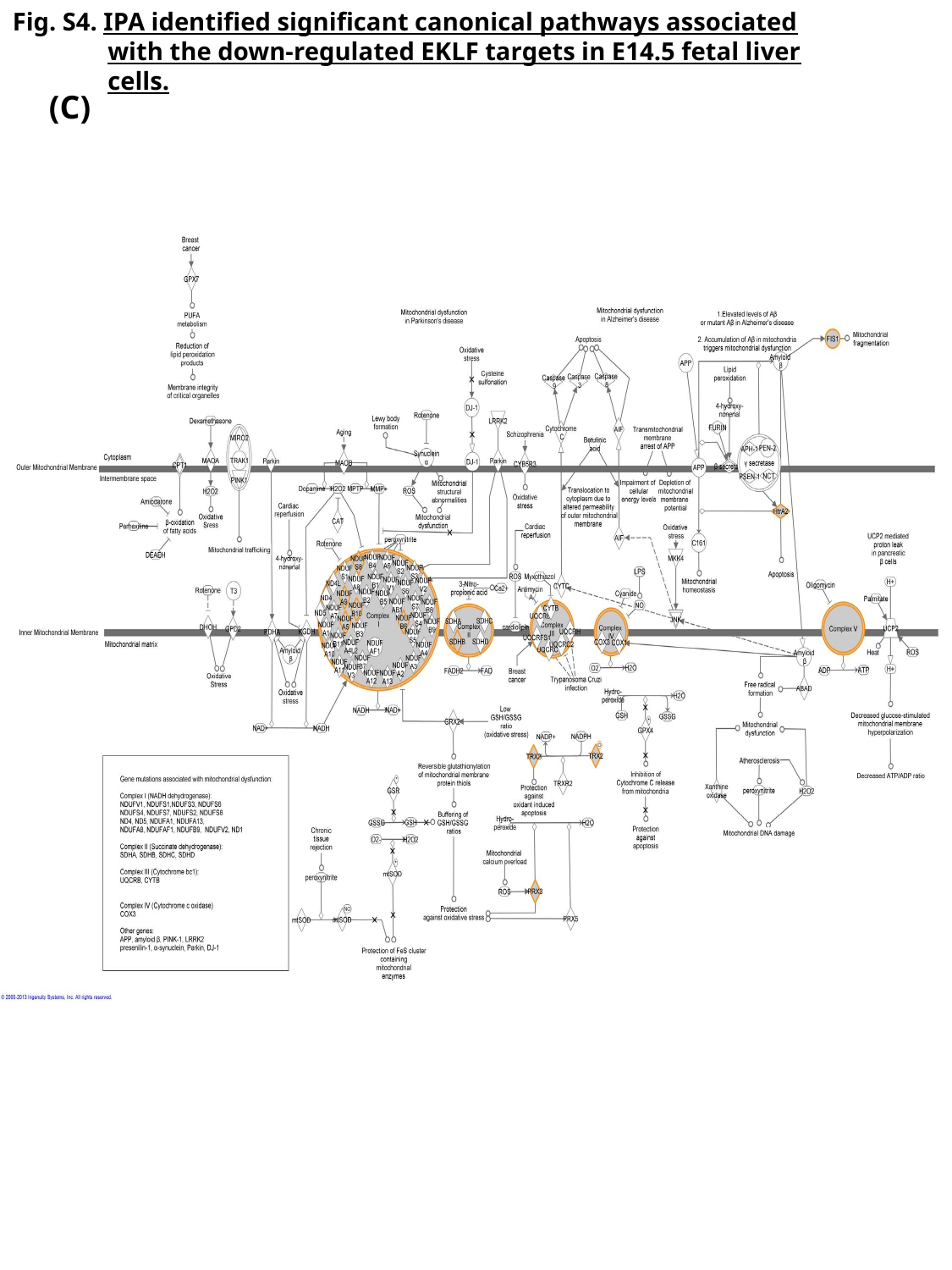

Fig. S4. IPA identified significant canonical pathways associated with the down-regulated EKLF targets in E14.5 fetal liver cells.
(C)

## Slide 16
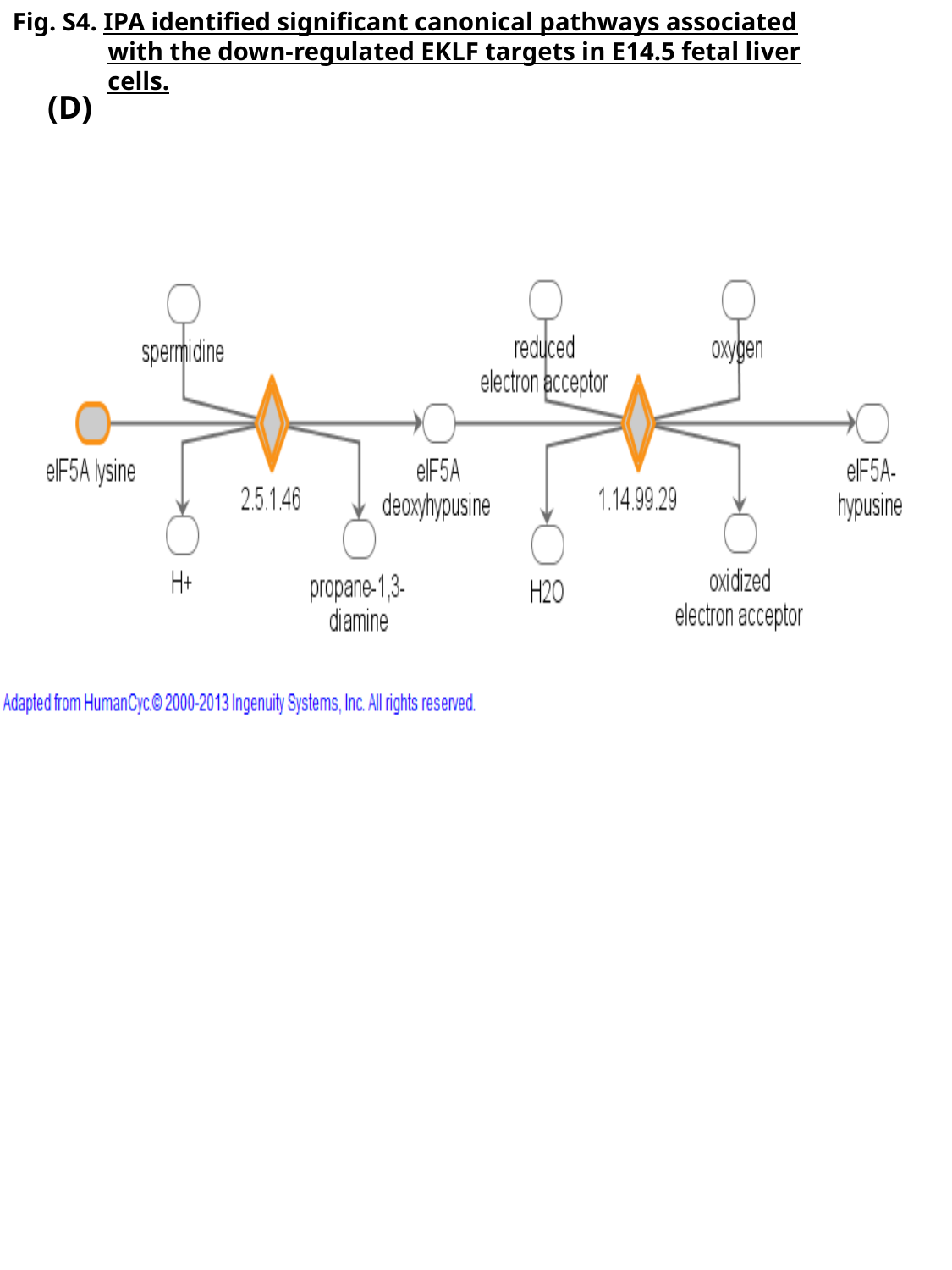

Fig. S4. IPA identified significant canonical pathways associated with the down-regulated EKLF targets in E14.5 fetal liver cells.
(D)

## Slide 17
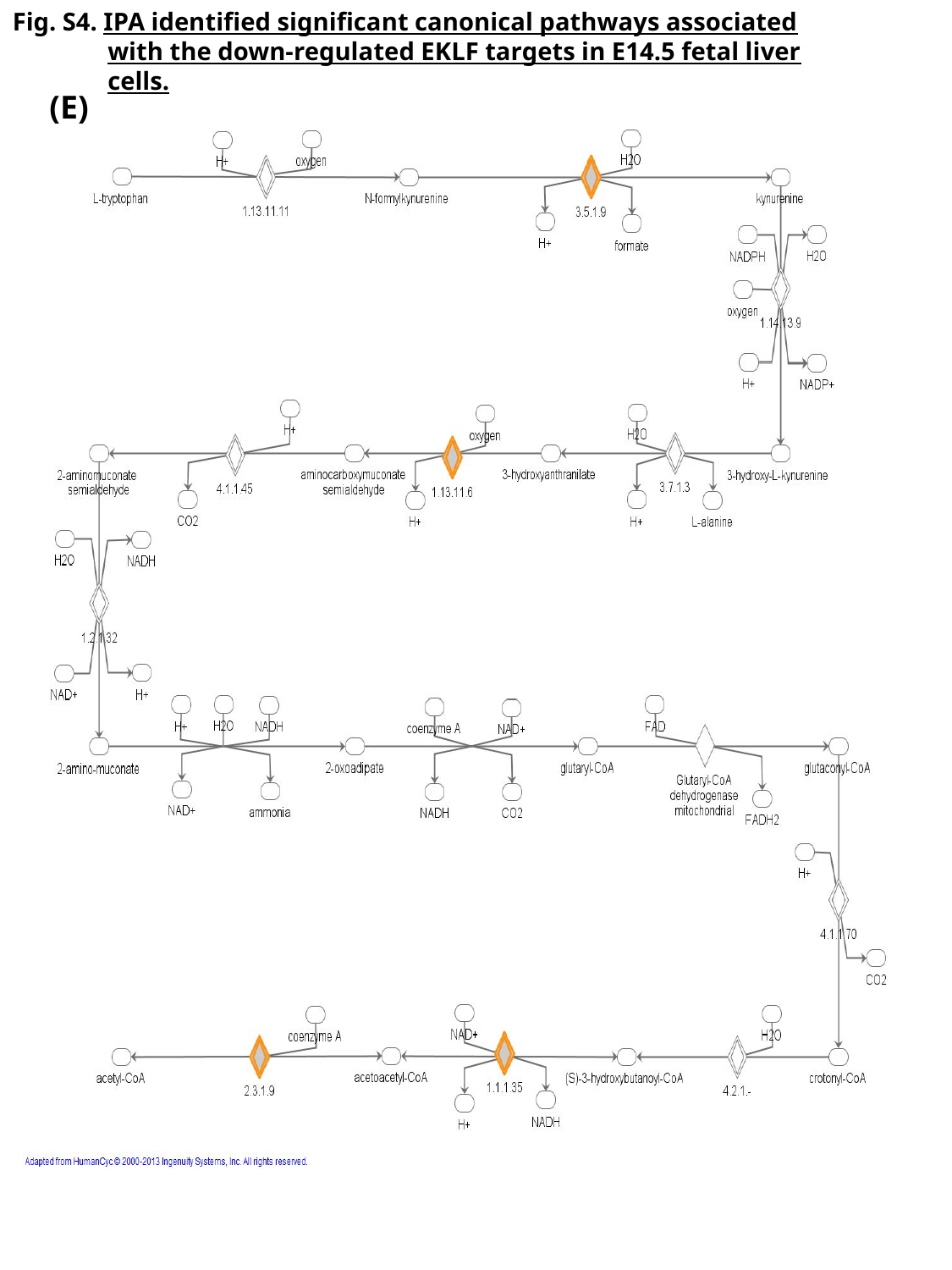

Fig. S4. IPA identified significant canonical pathways associated with the down-regulated EKLF targets in E14.5 fetal liver cells.
(E)

## Slide 18
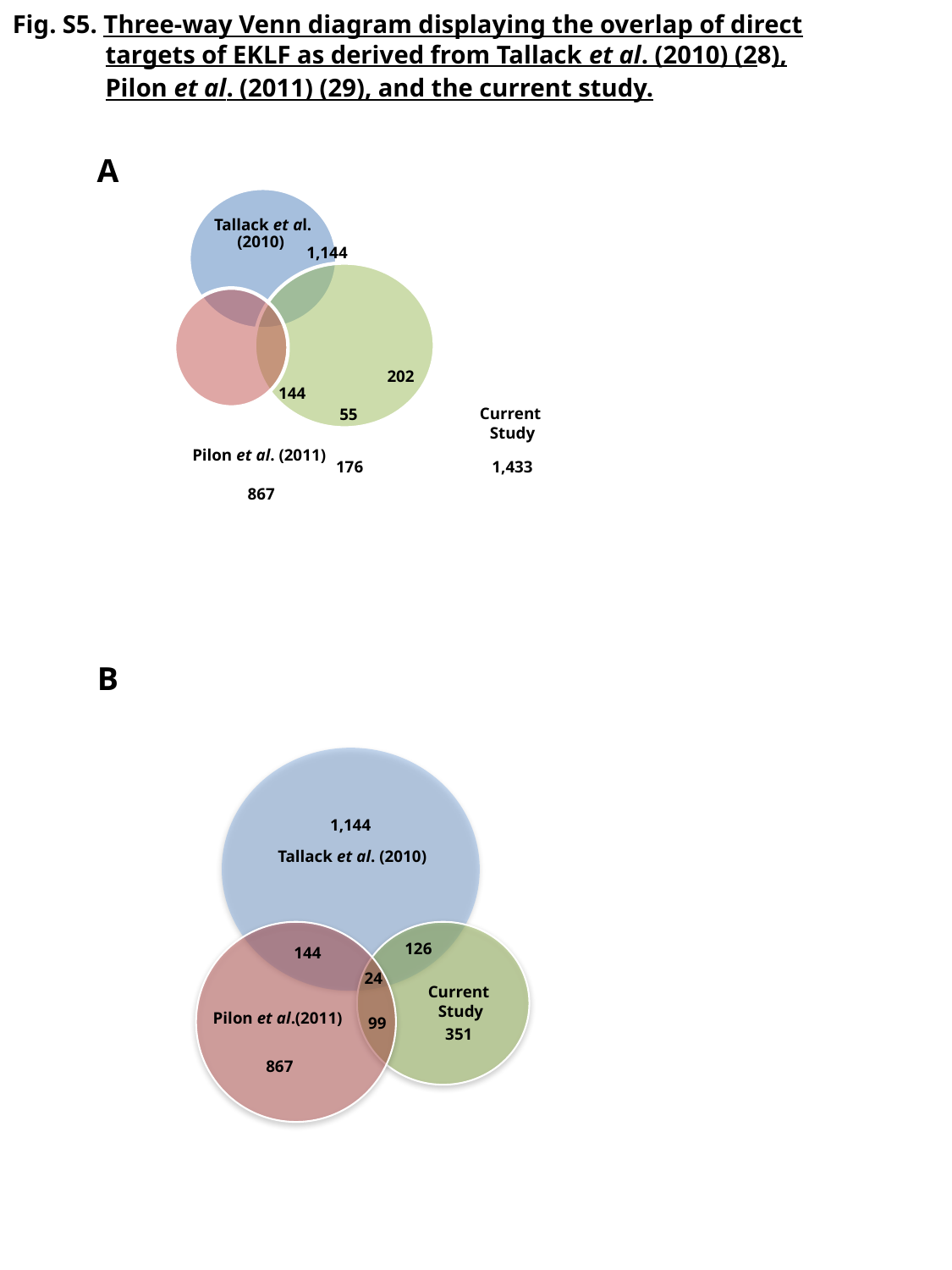

Fig. S5. Three-way Venn diagram displaying the overlap of direct targets of EKLF as derived from Tallack et al. (2010) (28), Pilon et al. (2011) (29), and the current study.
A
1,144
202
144
55
176
1,433
867
Current
Study
Pilon et al. (2011)
B
1,144
Tallack et al. (2010)
126
144
24
Current
Study
Pilon et al.(2011)
99
351
867
