# Supplementary material for: A Positive Regulatory Feedback Loop between EKLF/KLF1 and TAL1/SCL Sustaining the Erythropoiesis
Source: Int J Mol Sci. 2021 Jul 27;22(15):8024. doi: 10.3390/ijms22158024 (PMC8347936; doi:10.3390/ijms22158024)
Supplement: Supplementary file 1 [file ijms-22-08024-s001.zip › ijms-1297849-supplementary/Sup-table7-9.pdf]

Supplementary Table S7. List of PCR primers of used for validation of the ChIP-chip data.

ChIP-Q-PCR primers	Sequence 5' → 3'
Actb+668L	5'-GAC TAC CTC ATG AAG ATC CTG AC-3'
Actb+817	5'-CAG GGA AGA AGA GGA TGC GGC C-3'
βmajor-6411	5'-GTT CTC TGC ACA GAT AAG GAC AAA C-3'
βmajor-6623	5'-CTG ATC CTA CCT CAC CTT ATA TGC-3'
TAL1R-688	5'-CTC CCC GCG TGG GCT TTT GG-3'
TAL1R-475	5'-GTG AGG ATC ACA CCC GCC CG-3'
Tnfaip1 Dicer 1-F(2)	5'-GGT CTT GTA GTC TCT TTC CCG AT-3'
Tnfaip1 Dicer 1-R(2)	5'-ATC CGT GGT GCC TCC C-3'
Tnfaip1 Binding-F(2)	5'-GGG AGG CAC CAC GGA T-3'
Tnfaip1 Binding-R(2)	5'-CGG CTC TTA TGT TTC ACC CA-3'
Tnfaip1 Plasma 1-F(2)	5'-TGG GTG AAA CAT AAG AGC CG-3'
Tnfaip1 Plasma 1-R(2)	5'-AGC CAA GCA GAA CAA AGC G-3'
Tnfaip1 Dicer1-F(3)	5'-GGT CTT GTA GTC TCT TTC CCG AT-3'
Tnfaip1 Dicer1-R(3)	5'-ATC CGT GGT GCC TCC C-3'
Tnfaip1 Binding-F(3)	5'-GGG AGG CAC CAC GGA T-3'
Tnfaip1 Binding-R(3)	5'-AGC CAA GCA GAA CAA AGC G-3'
Tnfaip1 Plasma 1 -F(3)	5'-CGC TTT GTT TGC TTG GCT-3'
Tnfaip1 Plasma 1 -R(3)	5'-CAG ACC AGA TTC CAC CCA G-3'
NFE2 Dicer 1-F	5'-CAT CCC AAG GCA TCA GGC-3'
NFE2 Dicer 1-R	5'-TTC ACC CAG TCT ACC TCT CTT TGT-3'
NFE2 Binding-F	5'-ACA AAG AGA GGT AGA CTG GGT GAA-3'
NFE2 Binding-R	5'-CAG AAG ACC AGA GGA TAG GGC T-3'

ChIP-Q-PCR primers	Sequence 5’ → 3’
NFE2 Plasma 1-F	5’-AGC CCT ATC CTC TGG TCT TCT G-3’
NFE2 Plasma 1-R	5’-TCT CCT CAT CCT GCC TCC ATA-3’
E2F2 Dicer 1-F	5’-TCC CAG GCA GCG AAA CTC-3’
E2F2 Dicer 1-R	5’-CAA TGC TCT CAG TCA AGT CTC TCT C-3’
E2F2 Binding-F	5’-GAG AGA GAC TTG ACT GAG AGC ATT G-3’
E2F2 Binding-R	5’-GTC TGC TAT TTC ACT GGT CCC TTA-3’
E2F2 plasma 1-F	5’-TAA GGG ACC AGT GAA ATA GCA GAC-3’
E2F2 plasma 1-R	5’-GTG AGA GGC AGA GGC AGG TAG-3’
Xpo7 Dicer 1-F	5’-GAA GAG CAG AGC AGA GCC AGA-3’
Xpo7 Dicer 1-R	5’-TCC TTG GTG GCT CAG TCC C-3’
Xpo7 Binding-F	5’-GGG ACT GAG CCA CCA AGG A-3’
Xpo7 Binding-R	5’-AGT GAC AGT GAC CTC CTC CTC CT-3’
Xpo7 Plasma 1-F	5’-AGG AGG AGG AGG TCA CTG TCA CT-3’
Xpo7 Plasma 1-R	5’-TCC CTC TCC CTC TTT CTC CAA C-3’
PTAL1-5`D	5’-CTG GAT ACA GTA CAT AGA CAT TAA TAC-3’
PTAL1-3`D	5’-GCC AGA AGG TAG GTG TAT GTG-3’
PTAL1-5`P	5’-CTA CAA TGT ACC TAT GGG CTT C-3’
PTAL1-3`P	5’-CAT CCA ACT TCT CCC CAT TGG-3’
PTAL1-5`Non	5’-GCT GAA CGA GCA TCT GGG AGG-3’
PTAL1-3`Non	5’-GGA TGG CCC AGG AAT GCG CAG-3’
Csf2rb Dicer 1-F(1)	5’-CCC AGC AGC CTC AAG TTA TTC-3’
Csf2rb Dicer 1-R(1)	5’-CAG TAG GCA GTG GGA GCA GTC-3’

ChIP-Q-PCR primers	Sequence 5’ → 3’
Csf2rb Binding-F(1)	5’-GAC TGC TCC CAC TGC CTA CTG-3’
Csf2rb Binding-R(1)	5’-GAA ACA GAG AGC AGA TTG AGG AAG-3’
Csf2rb Plasma 1-F(1)	5’-CTT CCT CAA TCT GCT CTC TGT TTC-3’
Csf2rb Plasma 1-R(1)	5’-CAA GTC TGA GGA ATA CGC TAC CC-3’
Csf2rb Dicer 1-F(2)	5’-GAC TGC TCC CAC TGC CTA CTG-3’
Csf2rb Dicer 1-R(2)	5’-GAA ACA GAG AGC AGA TTG AGG AAG-3’
Csf2rb Binding-F(2)	5’-CTT CCT CAA TCT GCT CTC TGT TTC-3’
Csf2rb Binding-R(2)	5’-CAA GTC TGA GGA ATA CGC TAC CC-3’
Csf2rb Plasma 1-F(2)	5’-GGG TAG CGT ATT CCT CAG ACT TG-3’
Csf2rb Plasma 1-R(2)	5’-CCC ACT CAC CCT GTC CCA-3’
Fn3k Dicer 1-F	5’-CAT CTT AGT GTG AGG CTG GTC G-3’
Fn3k Dicer 1-R	5’-GCT CCT GCC TTC TCT ATG ACA A-3’
Fn3k Binding-F	5’-TTG TCA TAG AGA AGG CAG GAG C-3’
Fn3k Binding-R	5’-TGG GCA CAC ACA CAC ATA CAT A-3’
Fn3k Plasma 1-F	5’-TAT GTA TGT GTG TGT GTG CCC A-3’
Fn3k Plasma 1-R	5’-AGA GGC TAC TGT GGG AGG TGT-3’

Supplementary Table S8. List of RT-PCR primers of used for validation of microarray hybridization data.

Name	Forward primer	Reverse Primer
Dcbld2	5‘-CAAGGTGATGGATGTGGACAC-3'	5'-GCAGCATGGGTTCCACTCATG-3'
Pde4b	5'-TGGAAATCCTGGCTGCCAT-3'	5'-TCCACAGAAGCTGTGTGCT-3'
Hecw1	5'-AGCTGTCAATGCCTGTTCAG-3'	5'-TCCTCCTCCAAGCCATCTTC-3'
Nrip3	5'-AGGCTAAGTCTGAAGGGCTGAAG-3'	5'-TATCATCAACCACAGCTGCTTG-3'
STAT1	5'-AAGCGAACTGGATACATCA-3'	5'-CCGGGACATCTCATCAAAC-3'
JAK3	5'-ACTGCTGTTCGCTTGGCAGATC-3'	5'-CTCTGCCAGCAGCTCCAGGAG-3'
STAT2	5'-GGGACGAAGCTTTTGGGT -3'	5'-CTCTCGCCAGCCAACATT-3'
XK	5'-TGACGCTGCTCTTCTCCTTGATG-3'	5’-CTTCCAGTAGTGATGTTCTGCTCC-3'
IL12a	5'-TCAATCACGCTACCTCCTC-3'	5'-CAGAGCTTCATTTTCACTC-3'
IL16	5'-AACCGAGGACAGGAACCACT-3'	5'-CTTGAGAGATTTGCCATTGA-3'
Ankrd17	5'-TCCACCAGATGTCACTCAGCTAAC-3'	5'-GTACTGGCTGTCCAACTATCACTCC-3'
JAK2	5'-GAAGGCCAATGTTCTGAAAAAAG-3'	5'-ACTCCGTATCTGTAGGTTCTGCTG-3'
STAT6	5'-CAATGAGCCAGATGGGACCTTC-3'	5'-GGCTCTGAAATGAGTGGATGGA-3'
Tnfaip1	5'- CATGATCCGCGACGTGGAACT-3’	5'-GGCAGCCTTGTCCCTTGAAGA-3’
Ankrd1	5'-TGGCGATCGTGGAGAAGTTAATGG-3'	5'-GCGCACTCGTAATGACCAGTCCTC-3'

Supplementary Table S9. List of primers used for identification of *Tal1* exon-1.

Primer name	Sequence 5'→ 3'
Predict primer-F-1 (PF-1)	CGAAGACAGCCTGGTCTACAGAAG
Predict primer-F-2 (PF-2)	CAAGGCTACACAGACAAACCCTATC
Predict primer-F3 (PF-3)	CCAGTTTCCACCATCATAGAAAG
Isoform A exon 1 primer-R-1 (AR-1)	CGTATCTCAACACCCAGTTCCTCG
Isoform A exon 1 primer-R-2 (AR-2)	CAACCAACCCTCCCTTCTTCAT
Isoform A exon 1 primer-R-3 (AR-3)	CTACAAACAACCCGACTCGGATC
Isoform A exon 1 primer-R-4 (AR-4)	GATGCTCGTTCAGCGGCTTTG
